# Roaming in a Land of Milk and Honey: Life Trajectories and Metabolic Rate of Female Inbred Mice Living in a Semi Naturalistic Environment

**DOI:** 10.3390/ani11103002

**Published:** 2021-10-19

**Authors:** Paul Mieske, Kai Diederich, Lars Lewejohann

**Affiliations:** 1 German Center for the Protection of Laboratory Animals (Bf3R), German Federal Institute for Risk Assessment (BfR), Max-Dohrn-Straße 8-10, 10589 Berlin, Germany; kai.diederich@bfr.bund.de (K.D.); Lars.Lewejohann@bfr.bund.de (L.L.); 2 Animal Behavior and Laboratory Animal Science, Institute of Animal Welfare, Freie Universität Berlin, Königsweg 67, 14163 Berlin, Germany

**Keywords:** laboratory mice, animal welfare, environmental enrichment, behavior, physiology, individuality, housing condition, home cage monitoring, semi naturalistic environment

## Abstract

**Simple Summary:**

Different forms of environmental enrichment are used to increase the wellbeing of laboratory animals. These forms include extending the available cage space, housing a large group of animals within the same unit and adding stimulating physical objects. The semi naturalistic environment (SNE) used in this study implements all of these enhancements. However, there is debate as to whether such variation in housing standards increases the variability of experimental data. Indeed, it has been shown that mice living in the SNE developed individual differences in activity and behavioral parameters. Here, we investigated whether housing in the SNE enhances individual differences in aged animals and whether these differences are reflected in certain physiological parameters. These aspects were considered to assess the suitability of the SNE as a reference system in future studies. We found that the individual-level activity patterns of the animals stabilized during the housing period in the SNE. These behavioral characteristics did not correlate with the measured physiological parameters. Considering the variance of the measured data, which is comparable to the literature, the SNE seems to be a suitable system for studies comparing different housing systems in terms of animal welfare.

**Abstract:**

Despite tremendous efforts at standardization, the results of scientific studies can vary greatly, especially when considering animal research. It is important to emphasize that consistent different personality-like traits emerge and accumulate over time in laboratory mice despite genetic and environmental standardization. To understand to what extent variability can unfold over time, we conducted a long-term study using inbred mice living in an exceptionally complex environment comprising an area of 4.6 m^2^ spread over five levels. In this semi-naturalistic environment (SNE) the activity and spatial distribution of 20 female C57Bl/6J was recorded by radio-frequency identification (RFID). All individuals were monitored from an age of 11 months to 22 months and their individual pattern of spatial movement in time is described as roaming entropy. Overall, we detected an increase of diversification in roaming behavior over time with stabilizing activity patterns at the individual level. However, spontaneous behavior of the animals as well as physiological parameters did not correlate with cumulative roaming entropy. Moreover, the amount of variability did not exceed the literature data derived from mice living in restricted conventional laboratory conditions. We conclude that even taking quantum leaps towards improving animal welfare does not inevitably mean a setback in terms of data quality.

## 1. Introduction

Even in most well-planned and optimized studies using laboratory mice, the animals usually spend less time in the experiment than in everyday animal housing [1]. This should bring into focus the question of the suitability of the housing systems used. In Europe, minimal standards for animal housing are regulated by guidelines at national and international level (i.e., directive 2010/63/EU). Such guidelines stipulate, that mice should be kept in pairs or groups, taking into account sex-specific aspects to avoid undesirable reproduction or overly aggressive behavior. With regard to the size of cages the appendix of the EU directive gives minimum requirements of 330 cm^2^ in area (60 cm^2^ per mouse) and 12 cm in height. For reasons of costs and efficiency, most laboratory animal housing is based on these minimum criteria rather than on the space requirements that would be desirable from the animals’ point of view. Indeed, studies indicate that mice prefer to explore larger territories than provided in standard sized cages [2]. In addition, the EU Directive 2010/63 states that animals should be provided with ‘space of sufficient complexity to allow expression of a wide range of normal behavior’. In fact, it is questionable whether the minimum requirements meet this overall objective.

Although not always feasible in terms of maintenance and available laboratory space, a larger habitat would certainly mimic much more appropriately the ecological niche for which the mouse genome has evolved. Since mice are social animals, group housing during laboratory experiments is also important to promote animal behavior that comes as close as possible to their natural state. In addition, studies have shown that isolation of mice causes stress and lasting damage [3,4,5]. As a consequence, for biomedical research, a more natural setting might even strengthen translational research with regard to understanding how certain treatments affect mice without constraints imposed by barren housing conditions. In fact, natural enclosures have been successfully used to measure different kinds of behavioral, physiological and morphological parameters [6,7,8]. An enriched environment presented to a bigger group of animals may, however, increase the agonistic behavior within the group [9]. For example, in a study using a semi natural enclosure to characterize a transgenic model of Alzheimer’s disease [8,10], considerable numbers of agonistic interactions occurred between male mice. However, it is of note that the social structure and the behavioral patterns to establish such a mouse typical despotic social hierarchy could only be observed to the full extent precisely because the animals were kept in a large enclosure.

However, even when housed in small and barren cages, mice tend to exhibit individual personality-like traits leading to variability, which is seemingly resistant to even rigorous standardization [11]. In fact, the concept of animal personality has proved to be a viable way to explore variability in experimental data [12]. In order to understand the emergence of such individual differences, it has been a fruitful approach to observe the animals over long periods. In a previous study using a semi-naturalistic environment, it was shown that differences in exploratory behavior became more pronounced over time and correlated with adult neurogenesis and behavioral patterns [13,14]. In addition, repeatability of activity measures became increasingly stable during the early life of mice indicating that individual behavioral phenotypes were more predictable after adolescence [12].

In this explorative study, we focus on analyzing adult female mice regarding their activity, spontaneous behavior, and physiological parameters. Measurement of activity in our semi naturalistic environment is fully automated by the use of RFID antennas [8,10,13,14,15]. This creates a huge amount of continuous data without the need for handling or placing the animals into a novel environment. We examined the development of individual differences in a large group of twenty female C57Bl/6 mice with a special focus on the late adult phase of life. In contrast to previous studies, the mice were older (434 days) at the start of the activity measurement and the observation period also was longer (8 months). The unique dataset also allows the correlation of physiological and behavioral parameters as well as their activity patterns. Increased variability in the data due to housing the experimental animals in an enriched environment has already been addressed [16]. We aimed to determine whether a super-enriched environment increases the individuality of the mice across a variety of behavioral and physiological parameters. Thereby, we evaluate the suitability of the SNE as a housing system for comparative experiments possibly improving animal welfare in future experiments. Thus, we report on the life of these mice, which, in contrast to standard housing conditions, lived in a “land of milk and honey” with generous amounts of space to roam while having access to food and water *ad libitum*.

## 2. Materials and Methods

### 2.1. Animal Husbandry

When housed together with females, males are known to defend their territorial boundaries in such an enclosure [10]. In order to avoid pronounced aggression, we started by studying only females. Twenty female C57Bl/6J mice were purchased from Charles River (Charles River, Sulzfeld, Germany) at an age of eight weeks. At arrival the animals were special pathogen free and were then kept in standard cages in an open rack system. Our mouse facility conducts an annual health check in order to maintain the status. After seven days, the mice were tagged individually with a radio frequency identification (RFID) transponder. This was followed by another seven days of monitored recovery. During these two weeks, animals were kept in a home cage system consisting of two type IV cages connected by a plastic tube. At an age of 10 weeks, the animals were transferred to the semi naturalistic environment. Animals were habituated and kept at a 12/12 h light cycle (summertime lights on 8:00 a.m.–lights off 8:00 p.m., wintertime lights on 7:00 a.m.–lights off 7:00 p.m.), at 22.0 ± 2.0 °C, and 50.0 ± 5.0% humidity. Once a week, animals were weighed and handled to check for their health status. In the course of the study, one mouse died at an age of 404 days due to causes unrelated to this study, therefore the number of mice was reduced to 19 except for the weight development data. All experiments were conducted in accordance with the applicable European and national regulations and were approved by the State Office for Health and Social Affairs Berlin (G 0069/18).

### 2.2. Transponder Injection

All animals were marked individually for identification and activity measurement with an RFID transponder (ID 100, diameter: 2.12 mm; length: 11.5 mm, Trovan, Ltd., Douglas, UK). For pain treatment, 60 min before the injection, the animals received the non-opioid analgesic meloxicam (0.1 mg/kg, Meloxydyl, Ceva Tiergesundheit GmbH, Düsseldorf, Germany) orally. The transponder was subcutaneously injected between the shoulder blades under inhalation anesthesia with isoflurane according to established procedures (1.0–1.5% in 30% O_2_ with 70% N_2_O). The wound was then closed with tissue adhesive. The waking of the animals was monitored.

### 2.3. Semi Naturalistic Environment

**Figure 1:**
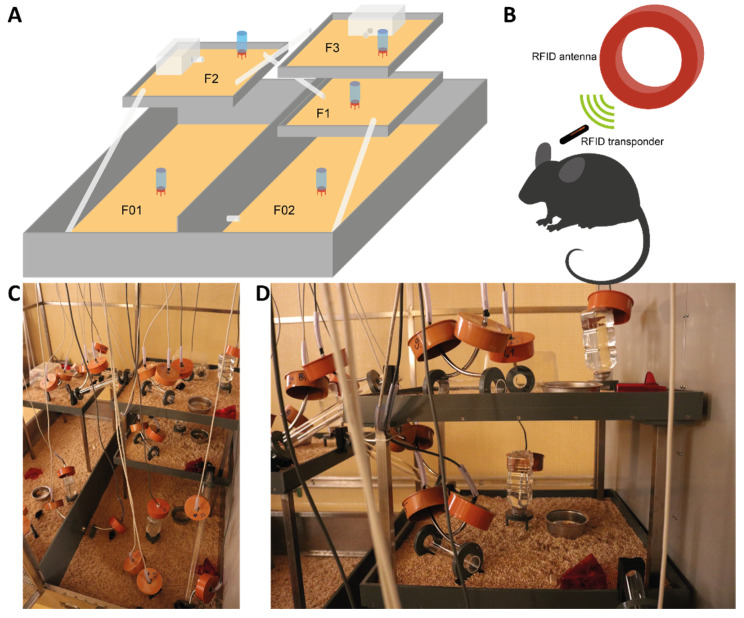
(**A**) Semi naturalistic environment (SNE) divided into five different levels (F01–F3). (**B**) Schematic illustration of the principle of tracking the mice through the RFID antennas. (**C**) Picture of the entire cage interior. (**D**) Zoomed in picture of the attachment of the antennas to enrichment tubes, drinking bottles and connecting tubes.

The semi naturalistic environment (SNE) is a large wire mesh cage of 1.7 × 1.7 × 2.1 m (length × width × height, Figure 1). The two-part base level and three elevated levels together create an area of 4.6 m^2^. The whole area was filled with aspen bedding (Polar Granulate 3–5 mm, Altromin, Lage, Germany) about 3 cm high. The different levels are connected with plastic tubes. Every level provides food (autoclaved pellet diet, LAS QCDiet, Rod 16, LASvendi, Soest, Germany) and water *ad libitum* and shelter for the animals. The two upper levels additionally provide two nesting boxes built from inverted type II cages with drilled-in air holes.

Throughout the SNE, 27 RFID ring antennas were placed systematically so that any change between the levels as well as the use of water sources and nesting boxes could be detected (Figure 1C,D). The arrangement of the antennas and the structure of the SNE is based on earlier work with a similar enclosure [15,17]. The antennas receive the signal of the transponder when a mouse passes through. The SNE was cleaned weekly by exchanging food, water, and soiled parts of the bedding. Every four weeks the whole cage was emptied and all surfaces as well as the nesting boxes and tubes were cleaned with hot water. During cleaning, animals were kept in a standard type IV cage with access to food, water and shelter as well as nesting material.

### 2.4. Measurement of Activity

Activity was measured as the number of contacts an animal had with the RFID antennas within the SNE. When a mouse passes an antenna, it receives a signal from the transponder of the animal. The antenna number as well as the unique ID of the animal is stored in a database with a corresponding timestamp. This was done with the Jerry 2 Recorder software used previously [15]. Thereby data of the movement and activity of every individual in the SNE was recorded continuously. To compare the spatial exploratory behavior, activity data was converted into the roaming entropy (RE). RE depicts a distribution value that sums the probabilities of every antenna being contacted by an animal at a certain time point (see Equation (1)). Thus, an animal that contacted many antennas on a broad range during an observed time obtains a higher RE than an animal that only passed a few antennas on a small area during the same time [13,14]. RE ranges between 0 and 1. By cumulatively adding up the RE for every observed time period (e.g., day, week), the cumulative roaming entropy (cRE) is gained (see Equation (2)).

RE and thus cRE have been calculated daily for the activity during the dark phase (12 h), since activity during the light phases was influenced by the possible presence of caregivers or the performance of experiments. It can be assumed that during the dark phase undisturbed behavior is shown and thus a higher comparability between the individual, observed time periods arises.

Equation (1): roaming entropy RE, i—individual, t—observed point of time or period of time, j—antenna, k—maximal number of antennas

$${\mathrm{RE}}_{i,t}=-\overset{k}{\underset{j=1}{\sum }}(p_{i,j,t}\log p_{i,j,t})/\log\left(k\right).$$

Equation (2): cumulative roaming entropy cRE, i—individual, t—observed point of time or period of time

$$\mathrm{cRE}={\mathrm{RE}}_{i,t_{1}}+{\mathrm{RE}}_{i,t_{2}}+{\mathrm{RE}}_{i,t_{3}}+\ldots +{\mathrm{RE}}_{i,t_{n}}.$$

### 2.5. Behavioral Observation

Spontaneous behavior of individually marked animals in the SNE was recorded by live focal animal observation. At the start of the observations, animals were 74 weeks old (524 days). Observations ended at the age of 80 weeks (565 days). Behavior was recorded in seven sessions of two days each scheduled between 7:30 and 9:00 a.m. Every individual animal was observed for 5 min per session. The order of observed animals was randomized. Behavior was recorded using the “Behavioral Observation Research Interactive Software” (BORIS) [18]. Short keys on a keyboard were pressed whenever a behavior occurred for frequency of behavior or pressed two times (start–stop) for duration of behavior. The time an animal spent resting (more than 10 s no movement) and time spent out of sight of the observer was subtracted from total observation time. The method for behavioral observations as well as the ethogram of the behavioral categories were based on Freund et al. (2015) [14].

### 2.6. Metabolic Rate

The principle of indirect calorimetry (TSE phenomaster, TSE Systems GmbH, Bad Homburg, Germany) was used to evaluate the metabolic rate. The calorimetry system was situated at a separate room at a 12/12 h light cycle, 22.0 ± 2.0 °C, and 50.0 ± 5.0% humidity. Animals were tested at the age of 82–84 weeks (584–594 days) in a randomized order. Following habituation to the experimental room (12 h), mice were weighed and placed individually in measurement cages equipped with bedding, shelter and nesting material. Food and water were accessible *ad libitum* during the entire measurement and were weighed before and after the experiment. Measurement cages and an empty reference cage were perfused with air. In the measurement cage, oxygen was lowered, and carbon dioxide was increased by the respiration of the animals during the measuring period (12 h light period). After flowing through both cages, the composition of air was compared between measurement cage and reference cage. By calculating the difference between air compositions, the metabolic rate of the examined animal was assessed. After the measurement the mice, food, and water were weighed, and the animals were placed back into their home cage. The resting metabolic rate (RMR) was measured as oxygen consumption rate ($\dot{V}O_{2}$) during the resting phases of the animals. To separate resting phases from active phases, the cumulative frequency percentage was plotted against the measured $\dot{V}O_{2}$. With a segmented linear regression, the threshold between $\dot{V}O_{2}$ of the resting phase and the active phase could be calculated. Data below the threshold were used to determine the RMR ([19]; R package ‘segmented’).

### 2.7. Timeline of Experiments

**Figure 2:**
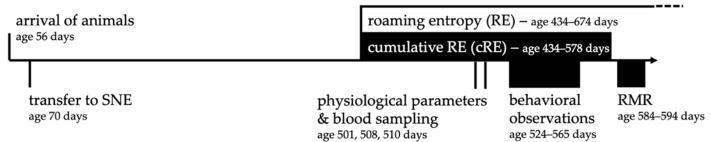
Schematic timeline of the housing of the experimental animals and the different experiments done.

After habituation, the experimental animals were kept for 364 days in the SNE before data acquisition was started. During this time the RFID setup was build, developed, and tested. Figure 2 shows the sequence of experiments performed during the housing in the SNE. At an age of 501 and 508 days, additional measurements of physiological parameters (bone density and grip strength of mice) were done as part of another study with a different focus. This also included a blood sampling on day 510. These data were not discussed in the present article but the fact that measures were conducted must be considered for the discussion of activity profiles.

### 2.8. Statistical Analysis and Calculations

Unless describes otherwise, all measured data is presented as mean ± standard deviation. In addition, the coefficient of variation (CV), the maximum value, the minimum value and the number of measured animals is given. The CV is calculated by dividing the standard deviation to the mean and is expressed as a percentage. It is used as an accepted parameter to evaluate variability within and across studies [20,21].

Analysis and illustration of data were done with the software environment R (v 3.6.3, R Foundation for Statistical Computing, Vienna, Austria, R Studio v 1.2.1335, RStudio, Inc., Boston, MA, USA). Continuous data (body weight and RE) were analyzed using linear models (‘lm()’ function). Related predictors were added as mixed effects to the regression models (package ‘lme4’ [22], ‘lmer()’ function). Subsequent statistical comparison of different models (‘anova()’ function) identified the factors affecting the continuous data. Observed behavior was counted and analyzed as frequencies. Behavioral categories were compared descriptively. The physiological parameters were measured once and therefore also evaluated descriptively and considered in a final correlation analysis. Correlation analysis was done with the function ‘cor.test()’. Multiple testing of individual parameters in a correlation analysis is a known source of alpha inflation and therefore a cautious approach for interpreting findings is recommendable. One approach would be to follow the Bonferroni method or the extended Bonferroni–Holm procedure to correct for multiple testing. However, a large number of correlations as it is common in exploratory research would lead to an overly conservative corrected alpha. Due to the exploratory design of the correlation analysis, we refrained from controlling for type I error by alpha correction but advice to interpret the calculated *p*-values with caution. To show a possible stabilization of the animals’ behavior in the SNE, the animals’ activity data in the form of RE were subjected to a repeatability analysis. The repeatability value R was calculated over a time period of four weeks (week 1–4). The following repeatability value R for direct comparison was also calculated over four weeks starting one week offset (week 2–5). This principle was continued until the end of the entire measurement period of RE. The repeatability was estimated with a linear model analyzing the mean RE depending on the individual animal as an influencing factor. The calculation of the repeatability was done with the R package ‘rptR’ and the function ‘rpt()’.

## 3. Results

**Figure 3:**
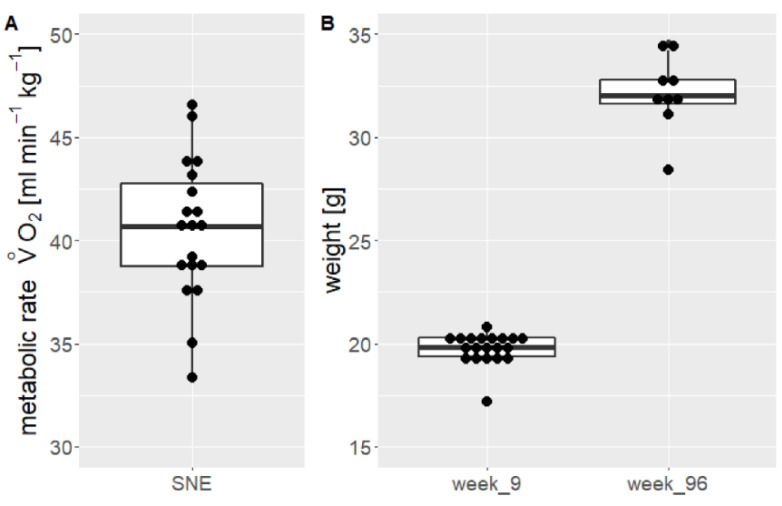
Summarizing boxplot of the resting metabolic rate (**A**) and the weight (**B**) of 19 female mice in the SNE. Metabolic rate was recorded at an age of 82–84 weeks. The weight is displayed at the animal age of 9 weeks and 96 weeks (start and end value).

Animals weighed 19.8 ± 0.7 g (CV = 3.5%, max = 20.8 g, min = 17.2 g, n = 20) at the time of arrival (Figure 3B). The weight of the animals increased significantly (F (1|1668) = 7.441, *p* < 0.0001, adj. R^2^ = 0.82). A mixed model applied to the data confirmed that weight increased over time and is influenced by the individual animal as a random effect and by time as a nested random effect. The applied model explains the variance of the data significantly better than a model that ignores time as a predictor (*p* < 0.0001). With 12–13 weeks of age experimental animals moved into the SNE. They stayed in this housing condition until the age of 96 weeks (670 days) and at this age a mean weight of 34.6 ± 3.0 g (CV = 8.7%, max = 40.0 g, min = 28.4 g, n = 18, Figure 3B) was measured.

### 3.1. Activity

Movement and activity data were recorded for 34 consecutive weeks, starting when the animals were 62 weeks (434 days) old. Therefore, the collected results reflect the condition and behavior of aged mice.

**Figure 4:**
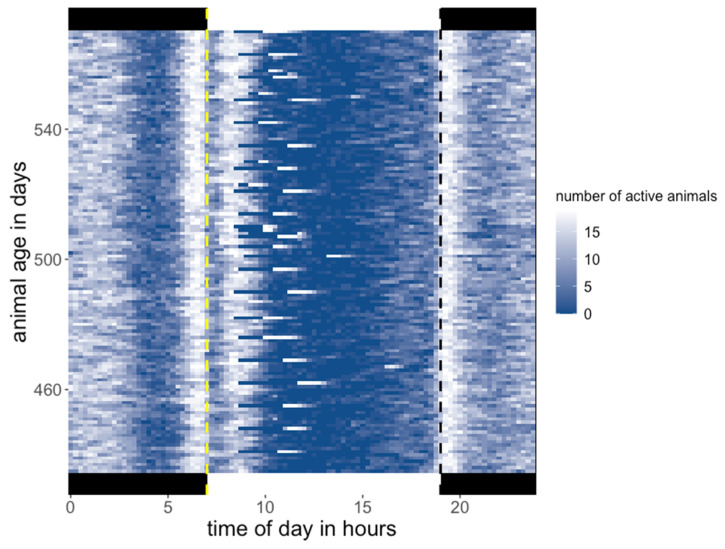
Heatmap of active animals in the SNE. Thy Y-axis shows the age of the animals in 136 consecutive days (day 434–day 570). Animals are recorded as active if there were at least a contact with two different antennas within a 15 min time period. The brighter a 15 min area, the more animals were recorded as active. The changing of the light/dark phase is marked by the dashed lines (yellow line = lights on, grey line = lights off).

Daily activity was comparable for all mice in the SNE. The activity pattern over the day is shown in Figure 4 on 136 consecutive days. During the night a constant high activity is shown until 3–4 h prior to the light phase. One hour prior to the light phase activity increases again until the resting phase of the animals that starts with the lights going on. The resting phase was interrupted by the presence of an animal caretaker, the weekly cleaning of the SNE, or experiments during the light phase. The rest of the light phase shows a low level of activity until it slowly increases again 1–3 h prior to the dark phase. The highest levels of activity are shown at the beginning and the end of the dark phase.

**Figure 5:**
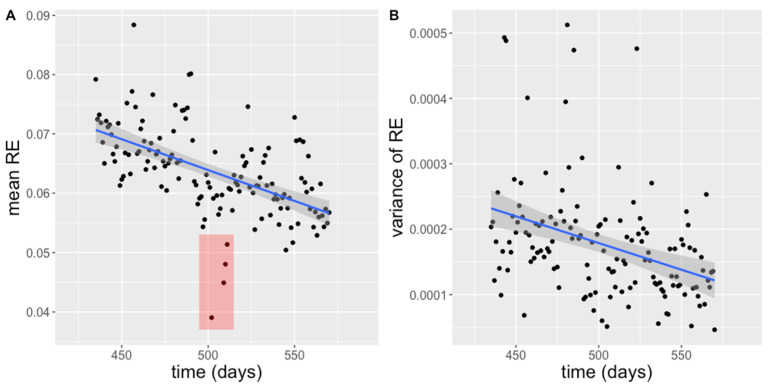
(**A**) Mean roaming entropy (RE) for 19 mice in the SNE over time with the change of the corresponding variance (**B**). The red area marks outliers in the values of the RE.

**Figure 6:**
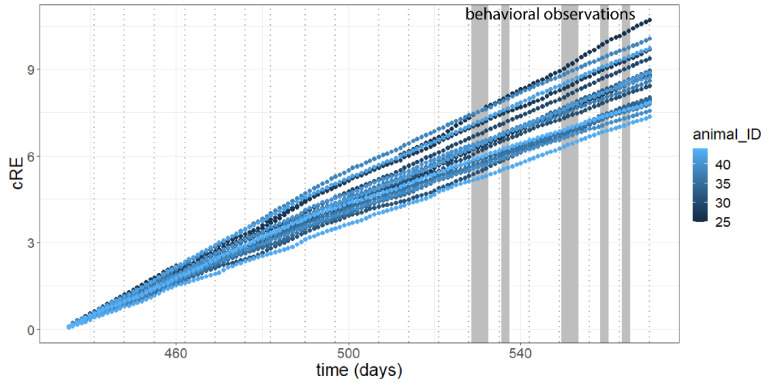
Cumulative roaming entropy (cRE) for 19 mice in the SNE over time. Vertical dotted lines indicate the weekly cleaning of the cage. Vertical grey areas indicate behavioral observations.

During the housing of mice in the SNE the roaming entropy could be measured for 236 days. The RE of the animals averaged 0.079 ± 0.014 (CV = 18.0%, max = 0.113, min = 0.059, n = 19, Figure 5A) on the first day and decreased over time (F (1|134) = 59.46, *p* < 0.0001, adj. R^2^ = 0.30) being influenced by the individual animal and time of measurement as random effects (mixed model). Including time as a predictor explains the variance of data significantly better than other applied models (*p* < 0.0001). The cumulative roaming entropy was calculated for 136 consecutive days (age 62–81.5 weeks or 434–570 days, Figure 6). At the end of the observed time period RE was 0.057 ± 0.007 (CV = 12.0%, max = 0.066, min = 0.042, n = 19, Figure 5A). The variance of the RE also decreased over time (F (1|134) = 29.76, *p* < 0.0001, adj. R^2^ = 0.13, Figure 5B). Although there was a decreasing range of RE for the animals, the cRE showed different slopes for different animals and thus seems to indicate individual differences in long-term activity (Figure 6).

The cRE increased to an average 8.654 ± 0.928 (CV = 10.7%, max = 10.716, min = 7.359, n = 19) over the time of 136 days (F (1|134) = 129100, *p* < 0.0001, adj. R^2^ = 0.99). In contrast to the variance of the RE, the variance of the cRE also increased over time (F (1|134) = 3342, *p* < 0.0001, adj. R^2^ = 0.96).

Figure 5A also showed outliers of the RE on the recording days 502, 509, 510 and 511. These minimum values were recorded in the nights after the measurement of bone density (day 501) and grip strength/blood sampling (day 508 and 510).

### 3.2. Spontaneous Behavior

**Figure 7:**
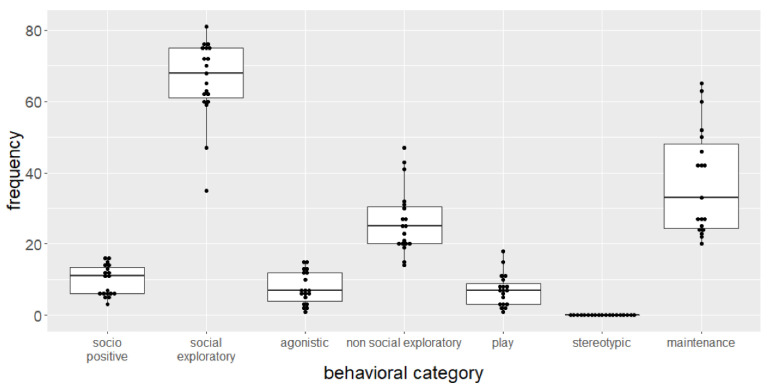
Mean frequency of behavior shown by female mice (n = 19) in the SNE during a cumulated 30 min observation period. Behavioral categories are combined from 28 possible behaviors to be observed.

Behavioral observations were completed with 19 animals. During the different observation periods some individuals were at rest or outside the field of the observer’s view. Therefore, seven behavioral observation sessions were conducted in total. Overall, no stereotypic behavior was observed (Figure 7). The highest frequency was shown in social exploratory behavior including approaching and sniffing a cage mate. Non-social exploratory behavior was observed at one-third of the frequency of social exploratory behavior. Socio positive, agonistic, and play behavior reached similar low frequencies. The second most observed behavior was maintenance behavior including drinking and feeding. If the categories are examined combined as self-related and social behavior, the animals showed more social behavior (83.26 ± 13.91 per 30 min) than self-related behavior (71.00 ± 17.99 per 30 min).

### 3.3. Metabolic Rate

The resting metabolic rate was measured for 19 animals. The mean weight of the animals at the time of the measurement was 34.7 ± 2.8 g (CV = 8.1%, max = 41.3 g, min = 29.4 g, n = 19, Figure 3). During the first 2–3 h of the measurement period, the metabolic rate fluctuated due to the animals exploring the cages and nearly reached maximum values for each individual. Since the measurement was performed during the light phase, the experimental animals eventually entered the resting phase. Therefore, 9–10 h of $\dot{V}O_{2}$ could be determined for each individual, interrupted only by brief increases in activity during which the animals ingested food or water or explored the cage before continuing to sleep. The mean $\dot{V}O_{2}$ was 40.5 ± 3.3 mL min^−1^ kg^−1^ (CV = 8.2%, max = 46.6 mL min^−1^ kg^−1^, min = 33.3 mL min^−1^ kg^−1^, n = 19).

### 3.4. Correlation Analysis

The results of the correlation analysis are summarized in Table A1 listed in the Appendix A. The metabolic rate did not correlate with the cRE. Weight showed a significant correlation with the cumulated activity at one of four examined time points (day 565, *p* = 0.0177). The overall weight change correlated with the decrease of RE over time (days 434–578, *p* = 0.00270). 

Furthermore, all observed behavior had negative correlation factors with the cRE. The more an animal interacted with a cagemate, operated maintenance or showed exploratory behavior the less antennas were passed. Only one significant negative correlation between socio positive behavior and roaming behavior was identified (*p* = 0.0297). All significant findings should be interpreted with caution as no correction for multiple testing was done.

## 4. Discussion

In the present study, behavioral observation, automated activity measurement and measuring physiological parameters provided full insight into the lifestyle and development of a group of female mice living in an SNE. The enriched housing system was suitable for keeping the test animals under laboratory conditions. All measurements could be performed as planned and provided results for an analysis of the emergence of individual differences in a large group of mice living in an SNE.

The overall activity of animals in the SNE decreased over time, which is common for aged mice [23]. Activity patterns during day and night were as expected [24] with higher activity during the dark phase compared to the light phase [25] and activity peeks around the time of daylight change. Previous studies using the SNE showed comparable behavioral patterns in female mice kept in a similar enclosure. The increasing variability in the cRE over time is also comparable to data from Freund et al. (2013) [13]. While in the beginning of the observation period cRE was similar between individual animals, individual differences in the daily RE led to increasingly divergent values of the cRE (Figure 6). After 136 days of observation, some animals displayed persistently higher spatial activity than other individuals of the group. This shows that these animals explored more enclosure space than others, or at least included more antennas in their routes through the enclosure. In contrast to the findings of Freund et al. (2015) [14] variance of cRE was found to be increasing, although variance of RE was decreasing over time. An increase in variability of overall cRE while the variability of the level of activity and roaming within the home-cage was getting lesser indicates a stabilizing behavior of the individual animals. Thus, we conclude that aged female mice each show individual routine roaming behavior.

**Figure 8:**
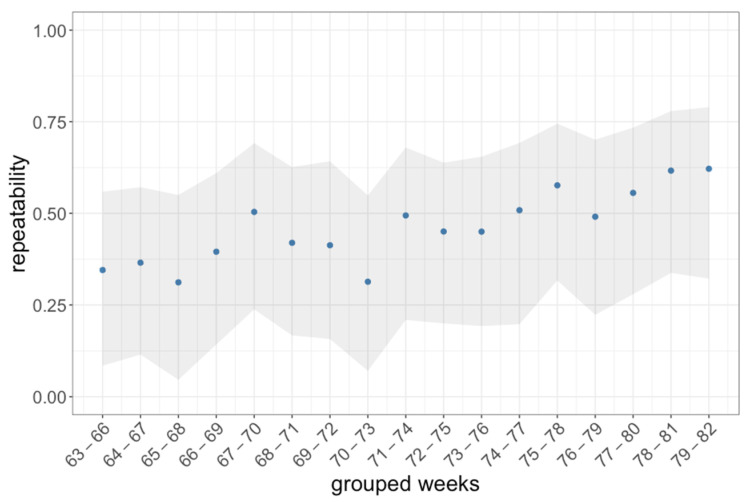
Calculated repeatability for the RE with the animal ID as a random factor. Each repeatability value (R, blue dots) was calculated over time periods of four weeks resulting in 17 groupings. The repeatability was estimated with a linear model. The grey areas depict the confidence intervals (2.5% and 97.5%) resulting from 500 bootstrapping runs and 100 permutations (R package ‘rptR’, function ‘rpt()’).

This stabilization of behavior over time was also shown by repeatability analysis of the RE (Figure 8). The repeatability value R was 0.34 at the beginning of the observation period and increased to 0.62 over time (F (1|15) = 37.08, *p* < 0.0001, adj. R^2^ = 0.69). This indicates that the behavior in the last four weeks is more stable on an individual level than in the first four weeks.

Figure 4 shows repeatedly a shift of the active phase that usually occurs in the morning to later in the day around early afternoon. This shift in activity could be attributed to the cleaning of the cage and handling of the animals on those days. Therefore, we checked whether the cleaning days cause effects on the activity in the respective following nights, which represent the determination periods for the RE. This was not the case, as the development of the cRE did not show a different course, if the nights following cleaning days were excluded from the calculation. Neither was the repeatability of the RE affected by the disturbance introduced through cleaning of the SNE.

It is of note that, unlike the unchanged cumulative activity values, the measurement system is sensitive enough to detect behavioral changes following potentially aversive experiments and interventions. In the RE, global minima were shown after measurements of physiological parameters associated with removal of the animals from the housing system and potentially aversive treatments. The reduced activity in the nights following these treatments was probably due to anesthesia during bone density measurement and the blood sampling that was conducted after the grip strength measurement. Just removing the mice from the SNE as it was done routinely during the cleaning did not lead to a reduced RE in the following night.

The second experimental focus was on spontaneous behavior of the animals. During the observation of spontaneous behavior, no stereotypical behavior was detected. The housing of 20 female animals within the SNE led to more social behavior than self-related behavior. Besides maintenance behavior, exploration of the environment and behavior directed to the cagemates were observed the most. The large area of the enclosure as well as the ability to keep a large group of animals allows the mice to perform a wide range of different behaviors. This is in contrast to small standard cages, where for example observing exploratory behavior would be challenging. The observed behaviors also correspond quite well to the natural behavior of mice and thus clearly shows the advantages of the SNE for the expression of the full range of the behavioral repertoire [10]. The observed behavior for a group of female mice might change if the SNE would house a group of male mice or a mixed sex group. Male mice show increased aggression, the bigger the available area and the bigger the group of animals [26]. Aggression between individuals would also increase with potentially pregnant animals [27].

The observations were also consistent with the findings of Freund et al. (2015) [14], where exploration was observed more frequently than other behaviors. However, non-social exploratory and play behavior occurred more often than social exploratory behavior in their data. Higher values for play behavior may be due to the younger age of the animals. The mice were observed at an age of six to twenty weeks. Play behavior is more common in such young animals compared to old mice [28]. In summary, in contrast to the results shown in the present study, Freund and colleagues observed more self-related behavior than social behavior. This difference could have been also caused by the different social structure in the group. Much older animals were examined in our study and a longer cohabitation could also favor a more pronounced social behavior.

Nevertheless, it is worth noting that the aged mice in the SNE showed any play behavior at all. Each individual performed sudden movements in vertical and horizontal directions with no apparent reason or interaction with a cagemate (e.g., chasing or being chased). These movement patterns are considered typical solitary play behavior. Play behavior in mice is normally found primarily in young animals, but play is enhanced by extremely enriched environments [28,29,30]. It is known that play behavior is most likely carried out in a positive life context while it is suppressed within unpleasant situations. Therefore, play behavior is deemed a suitable indicator of positive welfare that also applies to aged mice [31,32]. 

Since the SNE is a non-standard housing condition, we provide a detailed analysis of the data and a comparison with the literature in order to estimate potential flaws with regard to generalizability of results to more common housing conditions. The metabolic rate was obtained outside the home-cage and the described methods are widely used regardless of housing conditions.

Measuring the resting metabolic rate is dependent on a number of factors that strongly influence the results. Literature data vary to a considerable extent, and it is not always clear which factors have to be considered when comparing absolute values. To add to the confusion, there is no established standard with regard to the unit RMR is measured in. In order to evaluate our data against common findings from the literature we recalculated the measures given to the same units of measurement. Literature data on RMR ranges from 21.6 ± 1.7 mL min^−1^ kg^−1^ (CV = 7.9%) for female C57BL/6J mice [33] to 77.0 ± 6.3 mL min^−1^ kg^−1^ (CV = 15.4%) [34]. For male C57BL/6J mice in a study on the correlation of oxygen consumption to exercise values of up to 87.0 ± 4 mL min^−1^ kg^−1^ (CV = 4.6%) [35] were measured. Thus, our oxygen consumption rate of 40.5 ± 3.3 mL min^−1^ kg^−1^ is well within established findings, taking into account the differences in weight and age (Table 1). On the other hand, there are factors in the applied method that call comparability into question. The transport and measurement cages were typical transparent plastic cages and were therefore very different from the SNE. Due to the open mesh system, the animals were probably accustomed to different smells, temperature dynamics and ventilation characteristics. However, one major factor was probably the changed social constellation of the group during habituation and measurement. Since the calorimetry system does not allow measuring all animals at the same time, there is a division of the group for this time. Considering the sensitive social structure of these group animals, the isolation of a group member for the measurement must be considered as an important influence on the resting metabolic rate. Resting alone during the measurement also leads to increased thermoregulation and therefore also to an increased metabolic rate [36,37]. To evaluate the quality of our measurement further, we calculated the coefficient of variability (CV) as a way to compare the measures with regard to their prospective reproducibility. This analysis resulted in a CV of 8.1% for our RMR measurement, which is not at all conspicuous, compared to data from the literature. It is noticeable that measurements on the mouse group in the SNE generally led to low CV values, in some cases lower than in studies with standardized husbandry. Thus, if with regard to variability, comparable results can be obtained in a large and richly structured environment, the SNE could be used as a reference system in comparative studies. This is especially true if one aims at evaluating the physiological potential under more naturalistic conditions.

To further investigate the development of individual differences in the group, the results of the respectively performed measurements were correlated with the activity of the animals. Only few significant correlations were found. Roaming activity and weight of the animals correlated negatively. The less active an animal was within the SNE, the heavier it was. The correlation between body weight and activity in rodents is also suggested by studies on voluntary exercise [38,39,40]. Thus, although no alpha correction was made, we deem this effect as biologically meaningful. In addition, roaming entropy negatively correlated with socio-positive behavior. This may be explained by constraints of their time budget which would render actively roaming mice less likely to spent time interacting with conspecifics. However, Freund et al. (2015) did not observe significant effects of roaming and socio-positive behavior. Thus, we advise interpreting the relationship we observed with caution.

The metabolic rate did not correlate to the activity within the SNE or the weight of the animals. Overall, although our results show the development and stabilization of individual roaming behavior, these individual differences were not strongly reflected in the physiological parameters studied.

We are aware that the approach of studying a large group in a single SNE also comes with limitations. The aim was to determine whether the SNE would produce individual differences in experimental animals. If such individual differences exceed the variability found in conventional housing systems, it would be difficult to compare results in future studies using the SNE. We did not include a direct comparison of the SNE housed mice with mice from other types of housing but relate to literature data. However, we could demonstrate that the individual differences measured as CV did not exceed literature data. In addition, it is debated whether or not a single mouse or a group of mice housed in the same cage are to be considered as a statistical unit [41]. Some argue that individual results of all animals in one housing unit shall be averaged to only one statistical unit due to being mutually influenced by the same environment. However, here we analyze the effects of living in an SNE on the variability within the group. Living in an SNE deliberately includes living in a social environment. We therefore assume that any differences were caused by the influence of the housing and social system on the individual. Consequently, each experimental animal was treated as a separate data point.

In summary, the SNE represents a suitable housing system for a large group of female mice. The daily routine, such as the control and care of the animals, can be performed in a reasonable time compared to controlling conventional cage systems. In order to conduct physiological measurements, the mice have to be taken out of their familiar environment. Catching the mice was manageable in a reasonable time, however, still this procedure takes longer than just opening a standard sized cage. Thus, one concern might be that capturing, and transport affect the measured results more compared with standard housed mice. However, the results of this study do not confirm this assumption for the most part. Overall, the variability of the data is comparable to the literature. The handling of the animals outside the SNE was outweighed by the benefit of permanent monitoring of the home cage. The RFID system was able to record activity and thus behavioral data independently of light/dark cycles and experimenters and therefore enabled an unbiased understanding of mouse behavior in the SNE. These properties constitute the SNE as a powerful tool for phenotyping mice [42,43]. Finally, undisturbed long-term monitoring presents the opportunity to enhance reproducibility, as the most common cause of experimental variation is the experimenter.

## 5. Conclusions

In this study, the SNE was successfully used as an enriched housing system for a large group of female mice. Home cage monitoring was used to record the activity and observe the behavior of the animals. The semi naturalistic environment, as used and monitored here, promoted the emergence of individual differences with regard to the activity of the animals in the available space. The extensive recording and high sensitivity to external influences makes the roaming entropy the most important parameter of the SNE. The animals showed natural social and exploratory behavior, and even play behavior was observed in mice at a high age. Therefore, this housing truly follows the European directive (2010/63/EU) in providing ‘space of sufficient complexity to allow expression of a wide range of normal behavior’. The automated SNE is able to detect behavioral changes and did not prevent the taking of consistent measurements with sufficient accuracy. All in all, the SNE can be developed into an important tool for enhancing animal welfare in comparative studies.

## Figures and Tables

**Table 1:** Overview of the measured resting metabolic rate (RMR) with the associated coefficient of variability (CV) and the comparison to the literature values.

Source of Data	Sex of Animals	RMR in mL min^−1^ kg^−1^	CV in %
this article	f	40.5 ± 3.3	8.1
M. Konarzewski and J. Diamond, 1995	f	21.6 ± 1.7	7.9
T. D. Williams et al., 2002	f	77.0 ± 6.3	15.4
V. Schefer and M. I. Talan, 1996	m	87.0 ± 4	4.6

## Data Availability

The raw data supporting the conclusions of this article will be made available by the authors, without undue reservation.

## Appendix A

**Table A1:** Correlation results between measured parameters during the housing in the SNE and the recorded activity of 19 mice. The table shows the correlation factor r and the respective p-value below of each comparison made. Depending on an existing normal distribution the factor was calculated by the correlation methods according to Pearson or Spearman. Grey areas = *p*-values, that indicate a significant correlation between two parameters (uncorrected). If a parameter was used more than once for correlation analysis, the alpha mistake decreased by a n^−1^ according to Bonferroni correction.

Correlated Parameters	cRE (d578)	Weight (d502)	Weight (d510)	Weight (d565)	Weight (d578)	Weight Change (d434–d578)
cRE (d502)		−0.452				
		0.0520				
cRE (d510)			−0.418			
			0.0746			
cRE (d565)				−0.537		
				0.0177		
metabolic rate	0.0283				0.158	
	0.9086				0.518	
cRE (d578)					−0.45	
					0.0535	
slope RE (d434–d578)						−0.647
						0.0027
